# Relationship Between the Yo-Yo Intermittent Recovery Test and Match Running Performance in Canadian Male Professional Soccer Players

**DOI:** 10.3390/sports14020071

**Published:** 2026-02-06

**Authors:** Riccardo Bucciarelli, Farzad Yousefian, Ethan Brown, Lawrence Spriet, Margaret Jones, John Srbely

**Affiliations:** 1 Department of Human Health and Nutritional Sciences (HHNS), College of Biological Sciences, University of Guelph, Guelph, ON N1G 2W1, Canada; 2 Research Center in Sports Sciences and Human Development (CIDESD), Department of Sports Sciences, University of Beira Interior, 6201-001 Covilhấ, Portugal; farzad.yousefian@ubi.pt; 3 FPF Academy, Portuguese Football Federation, 1495-433 Oeiras, Portugal; 4 Faculty of Kinesiology, Sport, and Recreation (KSR), College of Health Sciences, University of Alberta, Edmonton, AB T6G 2H9, Canada; embrown@ualberta.ca; 5 College of Education and Human Development, George Mason University, Fairfax, VA 22030, USA; mjones15@gmu.edu

**Keywords:** association football, high-intensity running, global positioning system, multiple linear regression, performance analysis, sports recruitment

## Abstract

Despite the prevalence of the Yo-Yo Intermittent Recovery Tests Level 1 (YYIRTL1) and Level 2 (YYIRTL2) in elite soccer, knowledge surrounding their association and prediction of match performance is limited. This study investigated the association between respective tests and match running performance in male professional soccer players. High-intensity (HIR), high-speed (HSR), and sprinting (SPR) running distances were collected using a global positioning system from eleven professional male players who completed the YYIRTL1 and YYIRTL2. Associations between match performance and the YYIRT were assessed using correlational analyses, and the predictability of the YYIRT with match performance was assessed using univariate linear regression analyses. Strong correlations were found between YYIRTL1 and both HIR (r = 0.79) and HSR (r = 0.73). A moderate correlation was observed between YYIRTL2 and HIR (r = 0.42) and a weak correlation was observed between YYIRTL2 and HSR (r = 0.12). No correlation was observed between YYIRTL1 and SPR (r = 0.07) and a moderate, negative correlation was observed between YYIRTL2 and SPR (r = −0.21). Univariate regression analyses suggested that YYIRTL1 explained 63% of HIR variance, which YYIRTl2 did not, and that neither test suggested significant predictive ability in HSR or SPR. The YYIRTL1 is strongly associated with, and may predict, in-game HIR in Canadian male professional soccer players.

## 1. Introduction

Soccer performance imposes high physiological demands on athletes whereby contributions from the extramitochondrial (phosphagen and glycolytic) energy pathways are necessary to optimize performance in repeated high-intensity actions, and contributions from the mitochondrial energy pathway are necessary to optimize recovery between these high-intensity actions, during training and match play [1,2,3]. Recent developments in tracking systems technology, such as global positioning systems (GPS), have allowed the implementation of advanced time-motion analysis for the assessment of distances covered, as well as running velocities across different thresholds, during training and match play [4,5]. It has been well established that high-intensity activity performance variables, including high-intensity running (HIR; 13–15 kph; [6,7,8], high-speed running (HSR; 19.8–25.2 kph), and sprinting (SPR; ≥25.2 kph) distances [9] are valid measures for assessing match performance due to their associations with competition levels [10], as well as pivotal match-altering moments [11,12]. Such metrics have also been previously demonstrated to decrease throughout the match, which may indicate the onset of fatigue development related to increases in match tempo and pace [11]. Previous findings, therefore, highlight the importance of using fitness assessments to diagnose and predict players’ abilities to perform high-intensity activities during match play.

The Yo-Yo Intermittent Recovery Test (YYIRT) is a progressive and intermittent shuttle-run field test, commonly employed to evaluate athletes’ capacities to engage in high-intensity running during match play. The test imposes substantial demands on both the mitochondrial and extramitochondrial energy pathways [13], and performance of the test requires a running and activity pattern (including intermittent running at progressively increasing velocities with changes in direction, interspersed with rest periods nearly equal in time to the length of the running or work periods) which closely match the actual running and activity patterns seen in typical soccer match play [1]. For this reason, the YYIRT can be used to assist in both the recruitment and identification of soccer players, as well as to evaluate the effectiveness of training programmes. Two distinct versions of the test exist, including the YYIRTL1 and the YYIRTL2, and each version evokes different contributions from the mitochondrial and extramitochondrial energy pathways. The YYIRTL1 is strongly correlated with the mitochondrial pathway, with lower but still strong correlations to extramitochondrial glycolytic pathway [13]. In contrast, the YYIRTL2 exhibits a predominant relationship with the glycolytic pathway while still displaying substantial association with the mitochondrial pathway [13]. Both tests possess excellent test–retest reliability, reproducibility, and sensitivity to training in professional male soccer players [14], and they both serve as effective tools for discriminating amongst different competitive levels in professional male soccer [13].

The present study aims to extend existing evidence in three specific ways. Firstly, despite previously reported strong correlations between HIR and scores on both YYIRTL1 and YYIRTL2 tests [13,15], such studies defined HIR as total distance covered ≥15 km·h^−1^ and, thus, did not differentiate between HIR, HSR, and SPR velocities. The lack of research investigating the relationships between the YYIRTs and HSR and SPR match running distances represents a significant gap in the literature, as critical moments of the match, which may be crucial to the match outcome, have been reported to occur following sprints between opposing players contesting for the ball at high velocities [12]. Secondly, few studies have applied quantitative modelling approaches, even in an exploratory capacity, to evaluate the strength of association between YYIRT scores and match-derived performance metrics. Generating transparent effect-size estimates with appropriate uncertainty supports future hypothesis testing and sample-size planning. Thirdly, existing evidence has only been conducted using professional male European soccer players, while no such work has yet been conducted on professional Canadian players. Given the considerable differences between most European professional soccer leagues and the Canadian Premier League, including the colder Canadian climate, the shorter length of the Canadian competitive season, and the lower total number of matches played in Canada as a result of this shorter season—as well as the fact that at the time this research was conducted the Canadian Premier League was only in its third full competitive season, with the League comprising just eight professional clubs—the close replication of previous studies, utilizing a similar analysis of the relationships between YYIRT performance and match running performance, within the context of Canadian professional soccer, is warranted. Studying this context contributes novel, ecologically valid data from a hard-to-access professional population.

Therefore, the overall purpose of the present study was to explore and quantify the associations between performance on the YYIRTL1 and YYIRTL2 and in-match high-intensity running activities among professional Canadian male soccer players. Given the small, fixed population size typical of elite environments, and the context-specific nature of the sample, the analyses were conducted as an exploratory pilot aimed at estimating the magnitude and direction of these associations, rather than establishing definitive predictive models. Based on prior evidence from European professional cohorts, it was expected that YYIRTL1 would show the strongest association with HIR, while YYIRTL2 would relate more closely to HSR and SPR distances. The findings of this study provide initial effect-size estimates of these relationships in a previously unexamined professional context. From a pragmatist perspective, this work offers applied insights into how YYIRT performance reflects match-related running tendencies across multiple games, thereby offering contextually grounded insights that can guide practitioner decision-making in training, monitoring, and talent identification.

## 2. Materials and Methods

A cohort of eleven professional male soccer players (5 defenders, 4 midfielders, 2 forwards; age: 23 ± 2.1 years; height: 182.3 ± 7 cm; mass: 80.3 ± 10.4 kg) were recruited from a team competing in the Canadian Premier League between August and October 2022. Prior to the start of the study, participants provided informed written consent and performed a Physical Activity Readiness Questionnaire+ (PARQ+) to be eligible for the study [16]. Participants were excluded if they were injured or were unable to participate in high-intensity exercise, as indicated by the PARQ+. This study was approved by the university research ethics review committee (Research Ethics Board # 19-05-004) and conducted according to the Declaration of Helsinki.

The testing protocol of players was completed across four days of the competitive season. Positional match running data were collected within an 8-week competitive-phase window (4 weeks before and 4 weeks after testing). This rolling window reflects a pragmatic approach consistent with applied sport-science practice, where athlete monitoring relies on multi-match trends rather than single-match samples, which are highly context-dependent. Thus, the match metrics used here represent each player’s typical running demands during the competitive period surrounding fitness testing, rather than a single match’s potentially volatile output; therefore, each participant had at least 2 games to be included in analysis. The four-day protocol formed part of a broader testing battery in this cohort, including squat jump [17,18], drop jump [19], isometric mid-thigh pulls [20], Cunningham–Faulkner test [21], VO_2max_ [22], and load-velocity profile [23,24]. Because the present manuscript focuses specifically on the relationships between YYIRT performance and match running metrics, only YYIRTL1, YYIRTL2, and GPS-derived match data were analyzed and elucidated here. Brief descriptions of the additional tests are provided in the Supplementary Material to support completeness and replicability.

On the morning of day 1 testing, athletes completed a standardized warm-up protocol, followed by a battery of jump and strength test. The test battery used comprised tests which were already familiar to the athletes, as they had completed them in previous phases of the competitive season.

Yo-Yo Intermittent Recovery Test, Level 1 (YYIRTL1) and Level 2 (YYIRTL2): On day 2 of testing, participants completed the Yo-Yo Intermittent Recovery Test Level 1 (YYIRTL1) on an indoor turf soccer field. The YYIRTL1 is a standardized test to measure running performance. The YYIRTL1 consists of repeated 20 m runs back and forth between a starting, turning, and finishing line. Each run started and ended with an audio cue, with the time between each getting progressively shorter, necessitating increases in speed throughout the duration of the test. There was a 10 s active recovery between each run. When a participant failed to reach the finish line in time twice, the test was completed, and their total distance covered was recorded as the test result [15]. Scores on the YYIRT1 were recorded as distance covered in metres. On day 4 of testing, participants completed the Yo-Yo Intermittent Recovery Test Level 2 (YYIRTL2), also on an indoor turf soccer field. The YYIRTL2 follows a similar protocol to the YYIRTL1, with successive 2 × 20 m shuttle runs performed, followed by a 10 s recovery period; however, the running velocities in the YYIRTL2 are higher, making the test more glycolytic in nature [15].

Match Analysis: Match running performance was assessed using Polar Team Pro H10 devices (Polar Electro Oy, Kempele, Finland), equipped with 10 Hz GPS, 200 Hz tri-axial accelerometer, gyroscope, magnetometer, and a HR monitor [25]. GPS devices with a 10 Hz sampling frequency are valid and reliable for measuring linear and team sport running and sprinting [26]. Specifically, the Polar Team Pro devices demonstrate acceptable inter-unit reliability for total distance, a variety of running speeds, and provide overall valid and reliable data [26,27,28]. Each player wore the same tracking device throughout the season, to prevent inter-unit error, for every training and match session. Players were securely fastened with the device against their skin, positioned approximately above the xiphoid process—the landmark at the bottom of the sternum. Recording commenced upon immediate detection of a heart rate signal. To ensure an optimal connection, the electrode was moistened with water, adhering to the manufacturer’s instructions. Following each session, the data was downloaded from the units and analyzed using proprietary software (Polar Team Pro, Version 2.0, Kempele, Finland). For each player during each match, total distance covered at high-intensity (HIR; 15–19.79 kph), high-speed (HSR; 19.8–25.19 kph), and sprinting (≥25.2 kph) running velocities were collected. The performance variables and their corresponding thresholds were selected based on similar previous studies [9,11,12]. The pre-match warm-up period and any additional time added to each half was removed prior to analysis. Only outfield players who completed a minimum of 2 matches at ≥75 min of regulation played each, with a total of 13 competitive matches included in the analysis. This threshold is consistent with recent GPS-based match-analysis research, where minimum participation criteria of 70–75 min was applied to align with substitute players having different running demands, and most substitutes entering after approximately 70 min of play [29,30].

Consistent with the applied nature of this research and the logistical constraints of studying professional athletes, the analyses were conducted within a pragmatist framework, which prioritizes generating context-specific, actionable knowledge under real-world conditions. Given the reviewers’ request for re-analysis and the fixed, limited sample available, the study was necessarily treated as exploratory in nature. This framing acknowledges that the analyses were conducted after inspection of the data and that the sample size does not support confirmatory modelling. Accordingly, all statistical procedures were selected to provide descriptive estimates of effect magnitude and uncertainty rather than to establish definitive predictive relationships.

Our primary aim was to examine the association between match running performance variable including high-intensity (HIR; >15.8–19.8 kph), high-speed running (HSR; >19.8–25.2 kph), and SPR (>25.2 kph) distances with the YYIRTL1 and YYIRTL2 tests. Associations between YYIRT scores and each match running variable were examined using Pearson correlations for normally distributed data and Spearman rank correlations when normality was not met. Bias-corrected and -accelerated 95% bootstrap confidence intervals (10,000 resamples) were computed to provide robust uncertainty estimates given the small sample size. The following criteria was adopted to interpret the magnitude of the correlation (r, ±95% CI): negligible, ≤0.10; weak, 0.10 < r ≤ 0.20; moderate, 0.20 < r ≤ 0.40; strong, 0.40 < r ≤ 0.60; very strong, 0.60 < r ≤ 0.90; and almost perfect-to-perfect, 0.90 < r ≤ 1.00 [31].

To provide preliminary effect-size estimates for future research, simple linear regression models were fit for each combination of YYIRT variable (YYIRTL1 or YYIRTL2) and match running outcome (HIR, HSR, SPR), resulting in six one-predictor models. Given the small sample (*n* = 11), multivariable or multi-level modelling was not attempted to avoid overfitting. Model out-of-sample predictive performance (generalizability) was evaluated using leave-one-out cross-validation (LOOCV), with cross-validated R^2^ and root mean square error (RMSE) reported descriptively. All analyses were framed as exploratory and aimed at estimation, rather than formal hypothesis testing. Statistical analyses were conducted in Python (version 3.11).

## 3. Results

Table 1 shows the descriptive statistics for different variables assessed, including distances covered in HIR, HSR, and SPR, as well as the YYIRTs; Table 2 shows the correlation analyses between the YYIRTs and match running performance. Results from Pearson’s r correlations indicated that YYIRTL1 distance was very strongly correlated to HIR (r = 0.79, *p* = 0.003) and HSR (r = 0.73, *p* = 0.02), whereas YYIRTL2 distance was strongly but not significantly correlated with HIR (r = 0.42, *p* = 0.19), and weakly but not significantly correlated to HSR (r = 0.12, *p* = 0.72). YYIRTL1 was unrelated (r = 0.07) and YYIRTL2 was moderately, insignificantly, negatively correlated with SPR (r = −0.21, *p* = 0.51).

The univariate model with HIR as the dependent variable and YYIRTL1 as the predictor showed a substantial in-sample association (R^2^ = 0.63, *p* = 0.003). The cross-validated estimate was lower (LOOCV R^2^ = 0.52) but remained clearly positive, suggesting reasonable out-of-sample predictive performance within this small cohort. In contrast, the model using YYIRTL2 showed much weaker in-sample association (R^2^ = 0.17, *p* = 0.19) and a negative LOOCV R^2^ (−0.18) (Table 3). Negative LOOCV R^2^ values indicate that, once cross-validated, the model’s predictions were less accurate than simply using the sample mean. This reflects instability in the association under out-of-sample evaluation, which is common in small samples or for variables that show high contextual variability.

For HSR, YYIRTL1 demonstrated moderate in-sample association (R^2^ = 0.49, *p* = 0.01), but the association was much weaker when cross-validated (LOOCV R^2^ = 0.11), suggesting limited predictive stability. YYIRTL2 showed minimal in-sample association with HSR (R^2^ = 0.01, *p* = 0.72), and the LOOCV R^2^ was negative (−0.58), indicating that this model performed worse than a sample mean predictor under cross-validation (Table 4).

For SPR, neither YYIRTL1 nor YYIRTL2 accounted for meaningful in-sample association (both R^2^ < 0.10). Cross-validated R^2^ values were negative for both models (−0.45 and −0.31, respectively), indicating no stable association between YYIRT performance and sprinting distance in this sample (Table 5). Table 3, Table 4 and Table 5 present the statistics from the regression analysis models for HIR, HSR, and SPR according to the YYIRTL1 and YYIRTL2 predictor variables.

## 4. Discussion

The primary aim of this study was to examine the association between specific match running performance variables and the incremental field tests YYIRTL1 and YYIRTL2, including a univariate regression analysis to explore the potential ability of the YYIRTL1 and YYIRTL2 tests in estimating specific match running performance variables. Findings from our study demonstrated that the YYIRTL1 had very strong correlations with both HIR and HSR, but not SPR, while the YYIRTL2 showed a strong correlation with HIR, a weak correlation with HSR, and no correlation with SPR. Univariate regression analysis with cross-validation yielded results which indicate that the YYIRTL1 may be a stable, strong predictor of in-game HIR, but not HSR, while the YYIRTL2 is not, and finally, that neither YYIRT is predictive of in-game SPR.

Our findings build on the results from previous literature, which showed strong correlations between the YYIRTs and in-match HIR [13,16]. Further, the strong correlations observed between the YYIRTs and HSR in our study suggest that these tests may also be useful in predicting HSR; however, further research utilizing a larger sample is required before any definitive conclusions can be made. The use of univariate regression analysis as an exploratory method to examine the relationships between the YYIRTs and match-specific HIR and HSR performance represents a valuable tool to aid practitioners in the diagnosis of the physical abilities of male professional soccer players.

Although the YYIRTL1 and, to a lesser extent, the YYIRTL2 did show strong correlations with HIR and HSR, our univariate regression analysis was not able to demonstrate that either test can predict these in-game running performance metrics. From our analysis, the YYIRTL1 appears to have some predictive ability for HIR; however, the LOOCV R^2^ value was only 0.52, indicating that almost half of the variance in HIR performance is still unaccounted for with this model. While the YYIRTL1 does comprise an activity pattern very similar to that observed in competitive soccer match play, there may be one or more distinct physical abilities (e.g., the rate of change in velocity through accelerations and decelerations), which may not be directly related to the test, which in turn could help improve the ability of the test to predict HIR variance. Our models also failed to demonstrate: (1) predictive ability of the YYIRTL1 with HSR; (2) predictive ability of the YYIRTL2 with HIR or HSR; and (3) that neither of the YYIRTs was correlated to, or predictive of, in-match SPR. Therefore, players’ physical fitness capabilities, which may have stronger correlations and/or predictive ability of in-match HSR and/or SPR distance, remain to be established. Further research, including larger sample sizes and additional tests of physical fitness distinct from the YYIRTs, is warranted.

Professional male soccer players typically perform 150 to 200 high-intensity actions per match [10]. These actions are predominantly composed of brief explosive runs and sprints, which occur on average every 72 s [32]. Further, a considerable proportion of such actions have been reported to precede goal-scoring opportunities, which in turn have a significant impact on match outcomes [33]. Performance of, and recovery in between, frequent, intermittent high-intensity actions, as seen in professional soccer, requires significant contribution from both the mitochondrial, extramitochondrial glycolytic, and extramitochondrial phosphagen energy pathways [1]. Physical fitness assessments, which also require strong contributions from these energy pathways, and which can demonstrate strong associations with the actual high-intensity running performed in match play, are potentially useful to coaches and practitioners working in male professional soccer.

While the direct physiological assessment of VO_2max,_ conducted in a laboratory setting continues to serve as the gold standard for evaluating mitochondrial fitness among athletes, including soccer players [3], recent research has increasingly turned to different methods of measuring the capacity of both the mitochondrial and glycolytic energy pathways. Modric et al. (2021) found that running speed at the aerobic threshold was strongly correlated to HIR (14.4–19.8 kph) distance among professional male soccer players in central playing positions, and that running speed at the anaerobic threshold was strongly correlated to HSR (>19.8–25.1 kph) and SPR (>25.1 kph) distances among players in wide playing positions [34]. In another recent study, Altmann et al. (2018) reported several moderate to very strong correlations between different measures of mitochondrial and glycolytic fitness, and both total in-match running distance, average and maximal in-match running velocity, and the number of high-intensity runs (defined as a run at ≥4 m/s for at least 2 s, while reaching 5.0 m/s) [35]. These results suggest that the addition of laboratory-based measurements of mitochondrial and glycolytic fitness, using gas analysis and/or blood lactate analysis during an incremental treadmill test, may help to improve the ability to predict in-match HIR and HSR in male professional soccer players.

In addition to the laboratory-based assessments of mitochondrial and extramitochondrial fitness, more soccer-specific field-based measures of mitochondrial fitness have also increased in popularity amongst coaches and fitness coaches working in male professional soccer, owing in part to their ability to accurately and reliably assess the fitness of multiple athletes or an entire team, over the course of ~20–30 min of training time, and without the need for expensive and time-consuming equipment and technology [13]. Notably, the YYIRTL1 has gained prominence, especially for establishing connections between fitness levels and in-match physical performance [13,15]. Recent research, although not focused on assessing the predictive capacity of the YYIRTL1, has shown strong associations between both the YYIRTL1 specifically, as well as other field-based incremental mitochondrial endurance tests in general, and HIR during match play in male professional soccer players [36,37]. Similarly, our findings demonstrated that the YYIRTL1, which has previously been shown to be strongly correlated to mitochondrial fitness and VO_2max_ [13], is also strongly correlated with HIR and HSR, and may also be predictive of HIR, in competitive matches for professional male soccer players. The results of our study reinforce the findings of previous research, which has shown that both laboratory- and field-based tests of mitochondrial and glycolytic fitness are associated with in-match high-intensity running. The potential ability of the YYIRTL1 to significantly predict in-match HIR gives greater strength to the rationale for the use of this test as a diagnostic tool in male professional soccer.

Research utilizing comparable methodologies has also explored the role of the YYIRTs to predict in-match running performance among professional youth soccer players. One study found that the distance covered in the YYIRTL1 exhibited a moderate correlation with “moderate speed” running (8.01–13.00 kph) distance and accounted for 18% of the variance in match running performance [38]. Total distance covered in the YYIRTL1 has also been reported to exhibit a strong correlation with sprinting (r = 0.63), and both the YYIRTL1 and YYIETL2 demonstrated strong correlations with high-intensity actions (r = 0.56 and r = 0.57, respectively) [39]. When considering the results from research in both senior and youth male professional soccer, it appears as though the more soccer-specific field-based measures of mitochondrial and extramitochondrial fitness, like the YYIRTs, have stronger associations with in-match physical performance than laboratory-based measures. From these results, previous authors concluded that the continuous nature of the VO_2max_ test may lack specificity to the intermittent activity patterns observed in soccer, and this in turn may result in low correlations with running performance [39]. Since intermittent field-based tests like the YYIRTs comprise activity patterns which more closely mimic the actual running performed in soccer match play, it is logical that these tests have stronger associations with in-match running performance in soccer than continuous laboratory-based tests. While other intermittent versions of running-based VO_2max_ tests have previously been used, these tests have been shown to underestimate the true VO_2max_ of athletes [40] and are, therefore, a less popular method of assessing mitochondrial fitness. Findings from our study reinforce these conclusions, as we found that both the YYIRTL1 and YYIRTL2 tests had strong correlations with in-match HIR and HSR, and that the YYIRTL1 may have the ability to predict in-game HIR in male professional soccer.

Results from previous literature suggest that the use of the mitochondrial and glycolytic parameters derived from a continuous VO_2max_ test [34], as well as parameters derived via blood lactate samples during an incremental treadmill test [35], are the most effective ways to utilize laboratory-based assessments of mitochondrial and glycolytic fitness as a means of predicting match running performance. However, the use of intermittent field-based tests like the YYIRTs appears to be the most effective way to utilize more soccer-specific assessments of mitochondrial and glycolytic fitness, to predict in-match running performance in male professional soccer. Future research utilizing a combination of laboratory- and field-based testing, as well as additional performance variables, such as accelerations and decelerations into both correlational and regression models, may be warranted to determine whether these combinations may strengthen the associations or predictive ability of the YYIRTs with in-match HIR and HSR in male professional soccer.

To the best of our knowledge, the present study is the first to examine the relationship between the YYIRTs and in-match SPR using both correlations and univariate regression analysis in male professional soccer. Previous research has primarily centred on tests of laboratory-based mitochondrial fitness [34] and other tests [35], with limited attention directed towards the YYIRTs. Taken together, the results from previous research suggest that tests of physical abilities unique and distinct from the YYIRTs, including repeated sprint ability (RSA), maximal sprint velocity, and vertical jump height, may be better predictors of in-match SPR. Indeed, our own prior research determined that various measures of extramitochondrial phosphagen capabilities exhibited neither significant positive correlations with, nor predictive ability of, either the YYIRTL1 or the YYIRTL2. Physiologically, it is logical that the YYIRTL1 has shown strong associations with HIR and HSR, but not SPR. The running velocity thresholds for HIR and HSR used in our study (15–19.79 kph, and 19.8–25.19 kph, respectively) are speeds that any male professional soccer player can reach comfortably during match play [1]; therefore, the capacity to perform more HIR and HSR during match play is likely to be far more dependent on the mitochondrial and extramitochondrial glycolytic energy pathways, rather than the extramitochondrial phosphogenic system. Furthermore, the running velocities in the YYIRTs themselves also do not exceed 19 kph [15], and the YYIRTL1 comprises over 1600 m more running at lower velocities (14.5–16.5 kph) than the YYIRTL2 [16]. For this reason, the fact that the strongest association found in our study was between the YYIRTL1 (the test which requires participants to perform the greatest amount of running within the same threshold as HIR) and HIR itself is not surprising.

Because the YYIRTL2 primarily comprises running velocities ≥ 17 kph [16], it is unlikely to contribute more to the performance of HIR than the YYIRTL1; however, the fact that the YYIRTL2 showed a weaker correlation to HSR than the YYIRTL1 (0.73 versus 0.12, respectively) is surprising. Further research is required, but it may be the case that both the performance of and recovery between intermittent HSR during match play are more dependent on the mitochondrial rather than the glycolytic energy pathway, and as result, the YYIRTL1, which has been shown to be more strongly associated with the mitochondrial system than the YYIRTL2 [16], is the stronger correlate of HSR. Finally, since neither of the YYIRTs is positively associated with extramitochondrial phosphagen capabilities, it is not surprising that these same tests are also not associated with, nor can they significantly predict, in-match SPR performance. Future research should include both larger sample sizes and measures of extramitochondrial phosphagen capabilities as independent variables within a multiple regression model to determine which of these qualities may be predictive of in-match SPR in male professional soccer.

The findings of our study should be interpreted with several limitations in mind. First, the sample size was small (*n* = 11) and drawn from a single Canadian professional team. A sample of this size results in limited statistical power, so the findings reported in the study must be interpreted with caution as an exploratory pilot aimed at estimating the magnitude and direction of these associations between the YYIRTs and in-game running performance, rather than establishing definitive predictive models of these associations. The study of a Canadian professional team in the emerging Canadian Premier League may limit generalizability to other leagues, competitive levels, or cultural playing styles, as cultural disparities across nations may require adjustments in physical activity tailored to the style of each nation’s national league [41]. However, access to professional athletes is inherently constrained, and the present data offer context-specific evidence from a population that is rarely studied.

Second, match running data were drawn from an eight-week competitive-phase window surrounding testing. While this means the results cannot be tied to a single exact match-day fitness state, the design offers a practical advantage: averaging across multiple matches provides a more stable and ecologically valid representation of each player’s typical in-competition running demands than any single match, which is highly sensitive to contextual volatility (e.g., tactics, opposition strength, score-line, or weather). This approach aligns with applied practice, where coaches and sport scientists routinely interpret YYIRT performance in relation to multi-match trends rather than isolated snapshots.

Third, a range of plausible confounders (including playing position, tactical role, opposition strength, training load, psychological state, weather conditions, and broader allostatic load) could not be explicitly modelled here. With only 11 players, attempting to adjust for even a fraction of these would produce severely overfit and unreliable estimates. Instead, match data were averaged within players, which removes the nesting structure and provides a more stable representation of each athlete’s typical match running demands. This approach aligns with applied sport-science practice, where practitioners interpret YYIRT performance in relation to multi-match trends rather than single-match volatility. Future studies with larger multi-team samples will be needed to model these contextual influences using hierarchical designs.

Finally, recent research into in-match GPS-based running analysis in soccer has suggested that relative or player-dependent velocity thresholds may serve as a more accurate means of representing the running load experienced by players during match play, due to the high individual variance in acceleration and maximal running velocity abilities amongst high-level team sport athletes [42,43]. Future research should examine both standardized and individualized velocity thresholds within larger, multi-team datasets to determine whether thresholding methods meaningfully alter the relationship between YYIRT performance and match running outputs. With adequate sample sizes, more advanced modelling approaches such as multivariable regression, regularized methods (e.g., lasso, ridge), or tree-based models could quantify how YYIRT scores interact with positional, contextual, and load-related factors to predict high-intensity running demands more robustly than is possible in the present exploratory study.

## 5. Conclusions

Soccer requires high levels of various components of fitness; thus, having valid and reliable methods of determining how each of these components contributes to overall performance is critical to identifying which aspects of fitness require attention in training and athlete load management. Precise, valid, and reliable fitness assessments can help coaches and fitness coaches to better clarify the physiological characteristics of their athletes, and such assessments can also be used to predict the performance of high-intensity actions during match play and significantly impact the outcomes of match play. For practitioners aiming to develop and optimize the fitness of male professional soccer players, these assessments can serve as valuable tools for informed athlete recruitment and targeted training strategies. The findings of the present study demonstrate that the YYIRTL1 is very strongly correlated with in-match HIR and HSR and suggest that the YYIRTL1 may be able to predict HIR; that the YYIRTL2 is strongly correlated with HIR, and weakly correlated with HSR; and that neither test shows significant correlations with SPR. Taken together, these findings further demonstrate the external validity of the YYIRTs with regard to their association with fast running, but not sprinting, performance during match play. It is recommended that future research examines these relationships in larger samples of soccer players, as well as in other higher and more competitive levels of professional soccer. Future research should aim to utilize multiple regression or more advanced machine-learning models aimed at predicting in-match running. Finally, different measures of fitness, including speed, power, agility, and repeated sprint ability, should also be further explored with regard to their association with, and ability to predict, in-game sprinting. Our findings provide valuable insight to fitness coaches working with male professional soccer players in a scouting or player identification capacity.

## Figures and Tables

**Table 1:** Descriptive statistics of match running performance variables and YYIRTs.

Variable	Mean ± SD	95% CI Low	95% CI High
Total distance (m)	9959.61 ± 854.66	9385.44	10,533.78
HIR distance (m)	1268.49 ± 395.96	1002.48	1534.49
HSR distance (m)	479.60 ± 170.23	365.23	593.96
SPR distance (m)	151.22 ± 104.10	81.29	221.16
YYIRTL1 distance (m)	2778.18 ± 424.96	2492.68	3063.67
YYIRTL2 distance (m)	1309.90 ± 339.92	1102.54	1559.27

Descriptive statistics. Data presented as mean ± SD, with 95% Confidence Interval. Low and High to indicate the lower and upper bounds of the 95% confidence interval for the estimated parameter. Authors’ note: the small sample size (*n* = 11) results in limited statistical power.

**Table 2:** Correlational analysis between the YYIRTs and match running performance variables.

YYIRT Level	Pearson r Correlation (*p* Value) for HIR	Spearman Rank Correlation (*p* Value) for HIR	Pearson r Correlation (*p* Value) for HSR	Spearman Rank Correlation (*p* Value) for HSR	Pearson r Correlation (*p* Value) for SPR	Spearman Rank Correlation (*p* Value) for SPR
1	0.79 (*p* = 0.003)	0.69 (*p* = 0.01)	0.73 (*p* = 0.02)	0.63 (*p* = 0.04)	0.07 (*p* = 0.83)	0.10 (*p* = 0.77)
2	0.42 (*p* = 0.19)	0.46 (*p* = 0.15)	0.12 (*p* = 0.72)	0.31 (*p* = 0.35)	−0.21 (*p* = 0.51)	−0.09 (*p* = 0.79)

Correlational analysis. Table includes Pearson r Correlation Coefficients, and Spearman rank Correlation Coefficients, with *p*-values, between each YYIRT, and each match running performance variable.

**Table 3:** Regression analysis models for HIR.

Predictor	Slope	Standard Error	F-Statistic	95% Confidence Interval (Low, High)	R^2^	*p*-Value	β-Coefficient	LOOCV R^2^	LOOCVRMSE
YYIRTL1	0.740	0.188	15.421	0.484, 0.963	0.63	0.003 *	0.79	0.52	262.02
YYIRTL2	0.491	0.352	1.946	−0.110, 1.319	0.17	0.19	0.42	−0.18	410.13

Statistics from the univariate linear regressions using HIR as the dependent variable. Table includes Slope, Standard Errors, F-Stat, 95% Low and High Confidence Intervals, R^2^, *p*-values, β-Coefficients, Leave-One-Out Cross Validation (LOOCV) R^2^, and LOOCV Root Mean Square Error (RMSE). Significance set at alpha 0.05 and denoted by *.

**Table 4:** Regression analysis models for HSR.

Predictor	Slope	Standard Error	F-Statistic	95% Confidence Interval (Low, High)	R^2^	*p*-Value	β-Coefficient	LOOCV R^2^	LOOCVRMSE
YYIRTL1	0.2793	0.0957	8.521	0.045, 0.461	0.49	0.01 *	0.69	0.11	153.09
YYIRTL2	0.0601	0.1657	0.131	−0.221, 0.739	0.01	0.72	0.12	−0.58	204.49

Statistics from the univariate linear regressions using HSR as the dependent variable. Table includes Slope, Standard Errors, F-Stat, 95% Low and High Confidence Intervals, R^2^, *p*-values, β-Coefficients, Leave-One-Out Cross Validation (LOOCV) R^2^, and LOOCV Root Mean Square Error (RMSE). Significance set at alpha 0.05 and denoted by *.

**Table 5:** Regression analysis models for SPR.

Predictor	Slope	Standard Error	F-Statistic	95% Confidence Interval (Low, High)	R^2^	*p*-Value	β-Coefficient	LOOCV R^2^	LOOCVRMSE
YYIRTL1	0.017	0.0814	0.046	−0.183, 0.171	0.005	0.83	0.07	−0.45	119.73
YYIRTL2	−0.067	0.0995	0.454	−0.211, 0.276	0.04	0.51	−0.22	−0.31	113.82

Statistics from the univariate linear regressions using SPR as the dependent variable. Table includes Slope, Standard Errors, F-Stat, 95% Low and High Confidence Intervals, R^2^, *p*-values, β-Coefficients, Leave-One-Out Cross Validation (LOOCV) R^2^, and LOOCV Root Mean Square Error (RMSE).

## Data Availability

The data presented in this study are available on request from the corresponding author due to confidentiality requirements.

## Supplementary Materials

The following supporting information can be downloaded at: https://www.mdpi.com/article/10.3390/sports14020071/s1.

## Abbreviations

The following abbreviations are used in this manuscript:

YYIRTL1	Yo-Yo Intermittent Recovery Test Level 1
YYIRTL2	Yo-Yo Intermittent Recovery Test Level 2
HIR	High-Intensity Running
HSR	High-Speed Running
SPR	Sprinting
GPS	Global Positioning Systems
PARQ	Physical Activity Readiness Questionnaire
LOOCV	Leave-one-out cross-validation
RMSE	Root mean square error
kph	Kilometres per hour
RSA	Repeated Sprint Ability
VIR	Variance Inflation Factor
