# Supplementary material for: Relationship Between the Yo-Yo Intermittent Recovery Test and Match Running Performance in Canadian Male Professional Soccer Players
Source: Sports (Basel). 2026 Feb 6;14(2):71. doi: 10.3390/sports14020071 (PMC12944404; doi:10.3390/sports14020071)
Supplement: Supplementary file 1 [file sports-14-00071-s001.zip › sports-3974642-supplementary.pdf]

Supplementary Material

The testing protocol of players was completed across four days of the competitive season. Positional data were collected during nine competitive matches, occurring within two weeks prior to and two weeks following the 4-day experimental testing period. The four-day fitness testing protocol comprised several measures of fitness, including the YYIRTL1 and YYIRTL2 tests, an incremental treadmill-based VO_{2max} test, and a battery of jump and strength test, including squat jump, drop jump, and isometric mid-thigh pulls.

On the morning of day 1 testing, athletes completed a standardized warm-up protocol, followed by a battery of jump and strength test, including squat jump, drop jump, and isometric mid-thigh pulls. The test battery used comprised tests which were already familiar to the athletes, as they had completed them in previous phases of the competitive season.

Squat Jump: Athletes were instructed to maintain a static position on a force plate (Hawkin Dynamics, Illinois, USA) with a flexed-knee angle for 2 seconds before a jump attempt, without any preparatory movement, as high as possible, with no arm swing, and for three separate trials [1]. Athletes were not given any instruction about jumping technique, as previous research has indicated that subjects chose the depth that maximized both peak force and peak velocity resulting in maximal power output [2].

Drop Jump: each participant was instructed to drop down from a 42-centimetre box [3], landing on the force plate as fast as possible on the ground and then jumping up as high as possible, with no arm swing.

Isometric Mid-Thigh Pulls: Each athlete stood on the force plate with an Olympic barbell weighing 20.4 kilograms (45 pounds) pinned into a squat rack in front of them. The height of the bar was set to the mid-point of the thigh of each participant, while the participant's hands were holding the bar. Three maximal Isometric Mid-Thigh Pull trials were performed, with 60 seconds of rest between trials. Participants were instructed to pull as hard as possible on the bar for ~2 seconds until being told to stop. Maximal efforts commenced following a verbal countdown of "3, 2, 1, pull" [4].

Cunningham–Faulkner Anaerobic Test: In the afternoon of Day 1, athletes completed the Cunningham–Faulkner anaerobic test [5]. In this test, the treadmill was set at 8.0 mph (12.9 kph) speed and incline of 20%. The stopwatch started when the athlete began running unsupported. The test continued until the athlete could not maintain the speed required. Time was recorded to the nearest 0.5 seconds. Following completion of the test, participants performed a 15-minute cool-down. The same trained researcher performed all stopwatch testing to maximize reliability of test results.

Yo-Yo Intermittent Recovery Test, Level 1 (YYIRTL1): On day 2 of testing, participants completed the Yo-Yo Intermittent Recovery Test Level 1 (YYIRTL1) on an indoor turf soccer field. The YYIRTL1 is a standardized test to measure running performance. The YYIRTL1 consists of repeated 20-metre runs back and forth between a starting, turning, and finishing line. Each run started and ended with an audio cue, with the time between each getting progressively shorter, necessitating increases in speed throughout the duration of the test. There was a 10-second active recovery between each run. When a participant failed to reach the finish line in time twice, the test was completed, and their total distance covered was recorded as the test result [6]. Scores on the YYIRT1 were recorded as distance covered in metres.

VO_{2max}: On day 3 of testing, athletes completed VO_{2max} testing using a portable cardiometabolic testing system (PNOE Athens, Greece). This system has been validated against the COSMED Quark CPET, a previously validated stationary cardiometabolic testing system [7]. The test protocol comprised a 5-minute warm-up at a running velocity of 6 mph (9.5 kph) and an incline of 1% on a standard running treadmill (Woodway, Wisconsin, USA). Following warm-up, athletes completed a graded exercise test comprising 5 running stages, with each stage comprising running for 2-minute intervals, at progressively increasing running speeds, beginning at 8 miles per hour (12.9 kph), and increasing by 1 mph per stage, until a speed of 12 mph (19.3 kph) in the fifth and final stage.

Load-Velocity Profile and Theoretical Running Power: On day 4 of testing, participants completed 3 trials of a 30-metre linear sprint test using the 1080 Sprint (1080 Motion, Lindingo, Sweden) on an indoor synthetic turf field, with 2 minutes of rest between trials. The 1080 Sprint is a linear acceleration device that can be used to measure horizontal displacement and alter the load against which an athlete is moving via a cord and robotically controlled flywheel. The first run was performed with resistance set at the lowest possible load of 1 kilogram; the second trial was performed with a resistance of 8 kilograms; and the third and final trial was performed with a resistance of 15 kilograms, as per a previous protocol utilized by Thompson et al. (20220; [8]. For each trial, the athlete started behind a marked line in a standing split-stance and sprinted forward without any pre-start backward movement. When performing the resisted trials, athletes were instructed to "lean-in" to the harness to elicit higher starting forces. Athletes were instructed to be "as forward as possible" to eliminate any backward movement or countermovement that might affect their sprinting results [9]. Verbal encouragement was provided to ensure a maximal effort throughout each trial. Maximal running velocity (*Vmax*) was determined as the highest velocity (measured in metres per second, m/s) in a 5-metre segment of the 30-metre sprint.

Yo-Yo Intermittent Recovery Test, Level 2 (YYIRTL2); On day 4 of testing, participants completed the Yo-Yo Intermittent Recovery Test Level 2 (YYIRTL2), also on an indoor turf soccer field. The YYIRTL2 follows a similar protocol to the YYIRTL1, with successive 2x20-metre shuttle runs performed, followed by a 10-second recovery period; however, the running velocities in the YYIRTL2 are higher, making the test more glycolytic in nature [6].

References

1. Burr, J.F., Jamnik, V.K., Dogra, S., Gledhill, N. Evaluation of jump protocols to assess power and predict hockey playing potential. *J Strength Cond Res*. **2007**, *21(4),* 1139-1145. DOI: https://doi.org/10.1519/r-21496.1
2. Klavora, P. Vertical-jump tests: A critical review. *Strength Cond J*. **2000**, *22,* 70–75. DOI: https://doi.org/10.1007/s10195-017-0456-9
3. Flanagan, E.P., Comyns, T.M. The use of contact time and the reactive strength index to optimize fast stretch-shortening cycle training. *Strength Cond J*. **2008**, *30(5),* 32–38. DOI: https://doi.org/10.1080/14763140601058540
4. Haff, G.G., Ruben, R.P., Lider, J., Twine, C., Cormie, P. A comparison of methods for determining the rate of force development during isometric midthigh clean pulls. *J Strength Cond Res*. **2015**, *29(2),* 386–395. DOI: https://doi.org/10.1519/jsc.0000000000000705
5. Cunningham, D.A., Faulkner, J.A. The effect of training on aerobic and anaerobic metabolism during a short exhaustive run. *Med & Sci in Sports* **1969**, *1(2),* 65-69. NII Article ID: 10017180853
6. Krustrup, P., Mohr, M., Amstrup, T. The Yo-Yo intermittent recovery test: physiological response, reliability, and validity. *Med & Sci in Sports & Ex*. **2003**, *35(4),* 697-705.DOI: https://doi.org/10.1249/01.mss.0000058441.94520.32
7. Tsekouras, Y.E., Tambalis, K.D., Sarras, S.E., Antoniou, A.K, Kokkinos, P., Sidossis, L.S. Validity and reliability of the new portable metabolic analyzer PNOE. *Front in Sports & Act Liv*. **2019**, *1,* 24–24. DOI: https://doi.org/10.3389/fspor.2019.00024
8. Thompson, K., Safadie, A., Ford, J., Burr, J. Off-ice resisted sprints best predict all-out skating performance in varsity hockey players. *J Strength Cond Res*. **2022**, *36(9),* 2597-2601. https://doi.org/10.1519/jsc.0000000000003861
9. Cross, M.R., Lahti, J., Brown, S.R. Training at maximal power in resisted sprinting: optimal load determination methodology and pilot results in team sport athletes. *PLOS One* **2018**, *13(4),* e0195477. DOI: https://doi.org/10.1371/journal.pone.0195477
